# Evaluation of a self-management patient education program for patients with chronic heart failure undergoing inpatient cardiac rehabilitation: study protocol of a cluster randomized controlled trial

**DOI:** 10.1186/1471-2261-13-60

**Published:** 2013-08-23

**Authors:** Karin Meng, Gunda Musekamp, Bettina Seekatz, Johannes Glatz, Gabriele Karger, Ulrich Kiwus, Ernst Knoglinger, Rainer Schubmann, Ronja Westphal, Hermann Faller

**Affiliations:** 1 Department of Medical Psychology, Medical Sociology, and Rehabilitation Sciences, University of Wuerzburg, Klinikstr. 3, Würzburg 97070, Germany; 2 Rehabilitation Center Seehof, Lichterfelder Allee 55, Teltow/Berlin 14513, Germany; 3 Rehabilitation Hospital Heidelberg-Königstuhl, Kohlhof 8, Heidelberg 69117, Germany; 4 Rehabilitation Center Bad Nauheim - Rehabilitation Hospital Wetterau and Taunus, Zanderstr. 30-32, Bad Nauheim 61231, Germany; 5 Rehabilitation Hospital Kirchberg-Klinik, Kirchberg 7-11, Bad Lauterberg 37431, Germany; 6 Rehabilitation Hospital Möhnesee, Schnappweg 2, Möhnesee 59519, Germany; 7 Segeberger Kliniken GmbH, Rehabilitation Hospital, Am Kurpark 1, Bad Segeberg 23795, Germany

**Keywords:** Chronic heart failure, Patient education, Self-management, Evaluation, Cluster-RCT, Cardiac rehabilitation

## Abstract

**Background:**

Chronic heart failure requires a complex treatment regimen on a life-long basis. Therefore, self-care/self-management is an essential part of successful treatment and comprehensive patient education is warranted. However, specific information on program features and educational strategies enhancing treatment success is lacking. This trial aims to evaluate a patient-oriented and theory-based self-management educational group program as compared to usual care education during inpatient cardiac rehabilitation in Germany.

**Methods/Design:**

The study is a multicenter cluster randomized controlled trial in four cardiac rehabilitation clinics. Clusters are patient education groups that comprise HF patients recruited within 2 weeks after commencement of inpatient cardiac rehabilitation. Cluster randomization was chosen for pragmatic reasons, i.e. to ensure a sufficient number of eligible patients to build large-enough educational groups and to prevent contamination by interaction of patients from different treatment allocations during rehabilitation. Rehabilitants with chronic systolic heart failure (n = 540) will be consecutively recruited for the study at the beginning of inpatient rehabilitation. Data will be assessed at admission, at discharge and after 6 and 12 months using patient questionnaires. In the intervention condition, patients receive the new patient-oriented self-management educational program, whereas in the control condition, patients receive a short lecture-based educational program (usual care). The primary outcome is patients’ self-reported self-management competence. Secondary outcomes include behavioral determinants and self-management health behavior (symptom monitoring, physical activity, medication adherence), health-related quality of life, and treatment satisfaction. Treatment effects will be evaluated separately for each follow-up time point using multilevel regression analysis, and adjusting for baseline values.

**Discussion:**

This study evaluates the effectiveness of a comprehensive self-management educational program by a cluster randomized trial within inpatient cardiac rehabilitation in Germany. Furthermore, subgroup-related treatment effects will be explored. Study results will contribute to a better understanding of both the effectiveness and mechanisms of a self-management group program as part of cardiac rehabilitation.

**Trial registration:**

German Clinical Trials Register: DRKS00004841; WHO International Clinical Trials: = DRKS00004841

## Background

Chronic heart failure (HF) is a common, costly, disabling, and fatal medical condition encountered by a wide range of health care professionals in both primary and secondary care [1]. It is an illness that requires a complex treatment regimen over a life-long period. Therefore, self-care management/self-management is an essential part of successful treatment of patients with HF [2-6]. Meta-analyses/reviews provide evidence for the effectiveness of self-management interventions and patient education for HF-patients regarding knowledge, self-efficacy, self-management behavior, health-related quality of life, hospitalization and mortality e.g. [7-12]. However, studies show methodological shortcomings and further research is needed to determine independent effects of self-management interventions as well as different combinations of interventions [9].

Barlow and colleagues [13] described self-management as „the individual’s ability to manage symptoms, treatment, physical and psychosocial consequences and lifestyle changes inherent in living with a chronic condition, to effect the cognitive, behavioral and emotional responses necessary to maintain a satisfactory quality of life, so a dynamic and continuous process of self-regulation is established“. For HF patients, essential educational topics that should be covered include definition and aetiology, symptoms and signs, pharmacological treatment, risk factor modification, diet and exercise recommendations, sexual activity, immunization, sleep and breathing disorders, adherence, psychosocial aspects and prognosis, each associated with certain skills or self-management behaviors [3,5]. Thus, the focus on knowledge and providing information alone may not be sufficient, and guidelines recommend comprehensive HF education and counseling targeting skills and behavior [3-5]. Important research questions are evaluation of tailored programs, which includes specific educational strategies, identifying risk groups for poor self-care and effectiveness of innovative communication methods [4].

Generally, management of HF should be multi-professional and comprises several health care settings [14]. German guidelines recommend cardiac rehabilitation (CR) in particular for patients with HF caused by coronary heart disease or hypertension associated with specific educational needs and life-style changes [2]. CR in Germany [15] is predominantly offered as comprehensive inpatient treatment with a regular duration of three weeks. It is accessible for patients with myocardial infarction, surgery or catheter-based interventions after discharge from acute hospital care or patients with a chronic course of the disease and subsequent functional impairment. Although patient education is an essential part of CR [16] for HF, at the time of the conception of the study few standardized [17,18] and no evaluated educational group programs have been available for routine use so far. Moreover, many German patient education programs still lack certain quality requirements, such as the use of manuals, patient-oriented didactics, small-group format and evaluation of effectiveness [19,20]. Furthermore, theory-based techniques to foster health behaviors [21,22] are only rarely employed. In addition, few studies have compared different educational approaches applied within a multidisciplinary rehabilitation program in an inpatient setting e.g. [23-27]. Overall, further studies are needed to explore the effects of patient education programs, specific educational techniques and subgroups of patients who benefit most.

### Objectives

The aim of our study is to evaluate the short-, intermediate and long-term effects of a patient-oriented self-management educational group program as compared with a usual care program for HF patients receiving inpatient medical rehabilitation.

We hypothesize that the self-management education program is superior to usual care regarding self-reported self-management competence (primary outcome). In addition, we expect superior effectiveness of the new program regarding several self-management behaviors, such as symptom monitoring, physical activity, and medication adherence, as well as health-related quality of life and treatment satisfaction (secondary outcomes).

Moreover, moderator effects of (1) gender, (2) age, (3) education, and (4) type of rehabilitation - cardiac rehabilitation within 14 days after an acute cardiac index event (aCR) versus cardiac rehabilitation during the chronic course of disease without recent acute index event (cCR) - will be explored.

## Methods/Design

### Study design and data collection

The study is a multicenter cluster randomized controlled trial in four cardiac rehabilitation clinics. Clusters are patient education groups that comprise HF patients recruited within 2 weeks after commencement of inpatient CR. Participants will be recruited consecutively. In the intervention group (IG), patients will receive the new patient-oriented self-management educational program, whereas in the control group (CG), patients will receive a short lecture program (usual care).

**Figure 1:**
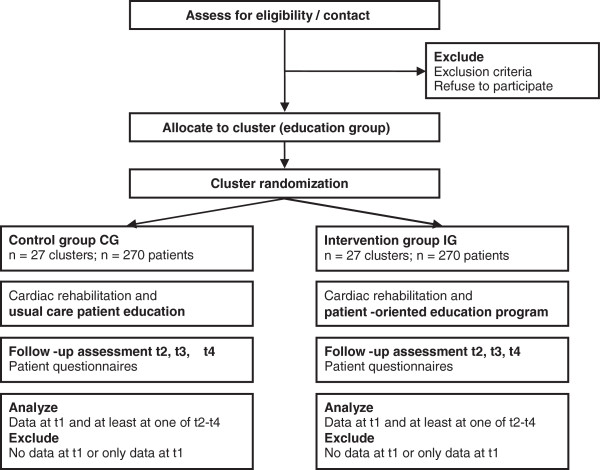
Study protocol diagram of data collection processes.

Data will be assessed at admission (t1) and three follow-ups, i.e. discharge (t2) and after 6 (t3) and 12 months (t4) using standardized patient questionnaires. Figure 1 shows the study protocol diagram.

Cluster randomization was chosen for pragmatic reasons, i.e. to ensure a sufficient number of eligible patients to build large-enough educational groups, to protect the blindness of the sample and prevent contamination by interaction of patients from different treatment allocation during rehabilitation.

### Ethical aspects

The study conformed to the principles outlined in the Declaration of Helsinki (http://www.wma.net/en/30publications/10policies/b3/17c.pdf) and was approved by the Ethics Committee of the Faculty of Medicine, University of Würzburg on 19 April 2011 (reference number: 60/11). Participation in the study is voluntary and based on written informed consent. Eligible patients will be informed about all relevant aspects of the study at the beginning of rehabilitation. Furthermore, they are informed of the right to refuse to participate or to withdraw consent to participate at any time without reprisal.

### Participants

Eligibility criteria for participants are a diagnosis of chronic systolic heart failure (ICD-10: I50), left ventricular ejection fraction (LVEF) of 40 or less, and New York Heart Association functional classification (NYHA) of class II or III. Exclusion criteria are acute events of decompensation, cognitive impairment, inadequate German language ability, and severe visual or hearing impairment.

All participants admitted to the rehabilitation clinic during defined time periods (algorithm defined for each clinic) will be eligible for building a cluster (education group), with a minimum of 3 participants per group as a prerequisite.

### Intervention

German inpatient multidisciplinary CR [15] includes medical treatment, exercise therapy/physical training, health education, psychological support, relaxation, and social counseling and comprises 3 weeks on average. In this trial, a self-management group program is compared to a basic medical lecture (usual care).

Intervention group (IG). Patients in the intervention condition will receive a self-management educational program “Curriculum Heart Failure” (for details see Additional file 1) that consists of 5 patient-oriented, interactive sessions of either 60 or 75 minutes each, which are provided in small groups of a closed format (15 participants or less). The program is manual-based and interdisciplinary with sessions led by a physician, a nurse, a psychologist and a physiotherapist, respectively. In each session, patients are actively involved in the educational process using a combination of didactic methods (short lectures, group discussions, practice, partner work and individual work). Didactic materials include presentations, flipcharts, and two patient booklets (educational booklet with HF information and worksheets, symptom-monitoring diary for twelve months). Contents of the lessons include HF illness and treatment knowledge (e.g. aetiology, symptoms and signs, diagnostics, treatment options such as non-surgical device treatment, surgery, and medication) with regard to individual information needs of the participants. Furthermore, the program focuses on self-management behaviors (e.g. dietary restrictions, attention to deterioration signs/symptoms, daily weight and blood pressure monitoring) and medication adherence. To promote physical activity, theory-based intervention techniques [21] are applied (e.g. action and coping planning, self-monitoring). Additionally, illness related problems in everyday life and signs of emotional distress with regard to HF and coping strategies are discussed.

The patient education program was developed by an interdisciplinary group of health professionals (members of the patient education workgroup of the German Society for Prevention and Rehabilitation of Cardiovascular Diseases, DGPR) and scientists with regard to previous work e.g. [17,18,27]. Both topics and didactics incorporate research evidence, guidelines, and quality criteria for patient-oriented educational programs. Patient acceptance and manual-based feasibility were verified in a pretest of 8 groups (2 in each participating clinic). Patients and trainers evaluated the education program with short standardized questionnaires directly after each session. Additionally, some sessions were observed and rated. Overall, results showed high acceptance and good feasibility. Based on open responses, small modifications were made to optimize the manual, intervention techniques and materials.

Control group (CG). Control condition is one lecture of basic medical education given by a physician with duration of about 60 minutes. Information is mostly presented in a vertical manner. Contents include basic HF illness information on aetiology, symptoms and signs, pharmacological treatment, non-surgical device treatment, surgery, and self-management recommendations (e.g. symptom monitoring and health behavior). Patients will receive three handouts which comprise main information on HF, and worksheets to list own medications as well as to monitor weight and blood pressure.

### Outcomes and measurements

We evaluate the effect of the patient education program on patient-reported self-management outcomes as well as health-related quality of life and treatment satisfaction. Therefore, all outcomes pertain to the individual level. The primary outcome is patients’ subjective self-management competence. Secondary outcomes include behavioral determinants and self-management health behavior (symptom monitoring, physical activity, medication adherence), health-related quality of life, and treatment satisfaction. Outcomes are assessed by standardized, validated measures. To assess symptom monitoring/control, a new measure will be developed based on existing instruments [28] and program-content. For details on outcomes, questionnaires and measurement points, see Table 1.

**Table 1:** Outcomes, measures, assessment

		**Assessment**			
**Outcomes**	**Measures**	**t1**	**t2**	**t3**	**t4**
Self-management competence (primary outcome)	Health education impact questionnaire heiQ, German version [29]. Subscales Self-monitoring and insight, Skill and technique acquisition	X	X	X	X
	Kansas City Cardiomyopathy Questionnaire KCCQ, German version [30]. Subscale self-efficacy	X	X	X	X
Determinants of behavior:					
Intention, action and coping planning, action control	HAPA-scales [31,32]	X	X	X	X
Medication beliefs	Beliefs about Medicine Questionnaire BMQ-D [33], German version	X	X	X	X
Self-management behavior:					
Symptom control	Questionnaire will be developed by our research group	X	---	X	X
Physical activity	Godin Leisure-Time Exercise Questionnaire [34], German modified version	X	---	X	X
Medication adherence	Medication Adherence Report Scale MARS-D, German version [35]	X	---	X	X
Health-related quality of life (disease specific)	Kansas City Cardiomyopathy Questionnaire KCCQ, German version [30]	X	X	X	X
Treatment satisfaction	Short questionnaire developed by our research group [36]	---	X	---	---

t1 indicates admission to rehabilitation; t2, discharge from rehabilitation; t3, 6 months after rehabilitation; t4, 12 months after rehabilitation.

Additionally, sociodemographic parameters (e.g. gender, age, marital status, education, occupation), as well as depression and anxiety (Patient Health Questionnaire, 4 item version PHQ-4 [37]), social support (ENRICHD Social Support Inventory ESSI [38]), smoking, and hospitalization after rehabilitation will be assessed. Medical data (e.g. diagnosis, NYHA, LVEF) will be provided by the attending physician.

### Sample size

We calculated the sample size based on expected effect size, power, and design effect (intracluster correlation, expected average cluster size [39]).

Sample size was powered to detect small to medium effects in the primary outcome in short-, intermediate and long-term (d = 0.3, 2-sided α = 0.05, 1-β = 0.8). Therefore, 352 persons were required. Furthermore, an intraclass correlation coefficient ICC of 0.02 and an average cluster size of 10 was assumed, resulting in a design effect of 1.18. By multiplication of the design effect and sample size without cluster effect, a necessary sample size of 208 patients in each group was obtained. Overall, we want to include 540 participants, based on an estimated drop-out of about 20%.

Power analyses comprise multiple uncertainties, i.e. ICC, actual cluster sizes, and effect sizes for primary and secondary outcomes. As the magnitude of the ICC varies with the venue of the trial and outcomes, no exact estimation of the ICC was possible because of the lack of data from previous studies reporting on ICC for psychological, behavioral and health outcomes for educational group clusters within inpatient medical rehabilitation. Table 2 shows an overview of possible parameter values (ICC, cluster size). Furthermore, power to detect smaller between-group effects on the secondary outcomes may be too low; thus, effect sizes will be reported throughout. For secondary outcomes, no adjustment for multiple tests is planned.

**Table 2:** Power of analysis of primary endpoint under different assumptions for the design effect

ICC	0.01	0.02	0.05	0.08	0.01	**0.02**	0.05	0.08	0.01	0.02	0.05	0.08
Cluster size	5	5	5	5	10	**10**	10	10	15	15	15	15
Design effect	1.04	1.08	1.20	1.32	1.09	**1.18**	1.45	1.72	1.14	1.28	1.70	2.12
Power (1-β)	0.85	0.84	0.79	0.76	0.83	**0.80**	0.72	0.64	0.81	0.77	0.65	0.55

Bold figures indicate: data for sample size calculation with expected effect size of 0.30 and resulting sample size n = 416.

### Randomization and allocation concealment

Clusters are randomly assigned to the two treatment conditions using a computer-generated list of random numbers. Randomization is performed by a scientific assistant of the research institute (central randomization per phone or e-mail) guarantying allocation concealment until a cluster has been recruited.

HF patients will be recruited at admission to inpatient CR and allocated to the next education group (cluster). Interventions will be performed every two weeks. Randomization results for the clusters will be communicated to the study assistants in each clinic per phone or e-mail only after building of a group and about two days before the intervention starts. Thus, patients will be recruited by physicians masked to later intervention allocation of the respective clusters.

### Blinding

Patients will be blind to the allocated study arm. Educational staff cannot be blinded due to their active role in treatment. Further therapeutic staff of the rehabilitation treatment may be blind to the allocated intervention.

### Statistical methods

All statistical analyses will be performed using SPSS for Windows. Prior to analysis, missing patterns will be explored. Missing data in accordance with missing at random (MAR) assumptions will be imputed using a multiple imputation procedure. Missing values due to drop-out will be analyzed by pair-wise deletion. Non-response-analyses and drop-out-analyses will be carried out by independent group comparisons using *t* tests for continuous variables.

The analysis of the primary and secondary endpoints will be done according to the intention-to-treat principle. Treatment effects (between-group effects) will be evaluated separately for each follow-up time point using multilevel regression analysis [39,40], with treatment group as fixed effects (IG, CG), individuals nested in clusters (education groups) as random effects, and adjusting for baseline values [41]. Statistical significance (p < 0.05, 2-sided) and effect sizes [42] will be reported for all between-group differences. Furthermore, ICCs for primary and secondary outcomes will be reported.

Moderator analysis will be performed by including the moderator variable as an additional fixed factor or covariate and examining interaction effects. Significance levels for interaction effects will not be adjusted due to their exploratory nature.

## Discussion

Patient education is recommended to foster self-management of patients with HF as several self-management interventions showed benefits on health outcomes. However, further research is needed regarding independent effects of single interventions as well as specific educational techniques and subgroups of patients who benefit most. In this cluster-RCT we will evaluate the short-, intermediate- and long-term effectiveness of a patient-oriented, self-management educational program as compared to a short lecture program (usual care) for HF patients receiving inpatient medical rehabilitation. Furthermore, subgroup-related treatment effects will be explored.

Methodological challenges of our study arise from evaluating a complex intervention as part of inpatient CR. Implementation requires training of different health care professionals and coordination with further multidisciplinary rehabilitation treatment. Furthermore, cluster randomization was chosen to ensure adequate numbers of eligible patients and to prevent contamination of the intervention. However, several uncertainties arise regarding the size of the design effect.

Altogether, study results will contribute to a better understanding of the effectiveness and mechanisms of a self-management group program as part of CR for a highly impaired illness population with a complex treatment regime.

## Abbreviations

BMQ-D: Beliefs about medicine questionnaire, German version; CG: Control group; CR: Cardiac rehabilitation; aCR: Cardiac rehabilitation within 14 days after an acute cardiac index event; cCR: Cardiac rehabilitation during the chronic course of disease without recent acute index event; DGPR: German society for prevention and rehabilitation of cardiovascular diseases; ESSI: Enrichd social support inventory; HAPA: Health action process approach; heiQ: Health education impact questionnaire; HF: Heart failure; ICC: Intraclass correlation coefficient; IG: Intervention group; KCCQ: Kansas City cardiomyopathy questionnaire; LVEF: Left ventricular ejection fraction; MARS-D: Medication adherence report scale, German version; NYHA: New York heart association; PHQ-4: Patient health questionnaire 4-item version; RCT: Randomized controlled trial.

## Competing interests

The authors declare that they have no competing interests.

## Authors’ contributions

KM is the principal investigator, developed the study, and is the main author of the manuscript. GM and BS undertook manualizing of the interventions, guidance of data collection and analysis and helped draft the manuscript. JG, GK, UK, EK, RS and RW are members of the patient education workgroup of the DGPR and contributed to development of the study and the intervention. They are listed as co-authors in alphabetical order. HF contributed to study development and helped draft the manuscript. All authors read and approved the final manuscript.

## Pre-publication history

The pre-publication history for this paper can be accessed here:

http://www.biomedcentral.com/1471-2261/13/60/prepub

## Supplementary Material

Additional file 1 **Description of the “Curriculum Heart Failure”.** Description of the intervention according to module, trainer, time, content, method, and material. Click here for file
