# Supplementary material for: Evaluation of a self-management patient education program for patients with chronic heart failure undergoing inpatient cardiac rehabilitation: study protocol of a cluster randomized controlled trial
Source: BMC Cardiovasc Disord. 2013 Aug 23;13:60. doi: 10.1186/1471-2261-13-60 (PMC3765303; doi:10.1186/1471-2261-13-60)
Supplement: Additional file 1 — Description of the “Curriculum Heart Failure”. Description of the intervention according to module, trainer, time, content, method, and material. [file 1471-2261-13-60-S1.pdf]

**Additional file 1 - Description of the "Curriculum Heart Failure"**

| **Module (Trainer)** | **Time (min)** | **Content** | **Methods** | **Materials** |
|---|---|---|---|---|
| Module 1: Heart failure – what does it mean and how can it be treated? (Physician) | 60 | Introduction to the program (content, targets, organization); HF: aetiology, symptoms and signs, diagnostics, treatment options, non-surgical device treatment, surgery, pharmacological treatment | Interactive short lectures, group discussion, homework | ppt-presentation, flipchart, educational booklet with HF information and worksheets ("the causes of my illness"), symptom-monitoring diary with worksheet ("my medication report") |
| Module 2: Heart failure – what can I do on my own? (Nurse, physician) | 75 | Lifestyle and self-care recommendations for cardiac patients and HF patients; symptom-monitoring; fluid balance; management of fluid and sodium intake; signs and management of fluid overload and deterioration; daily weight, pulse and blood pressure monitoring | Interactive short lectures, group discussion, individual work and homework | ppt-presentation, flipchart, educational booklet with HF information and worksheets ("my individual recommendations for a healthy life", "how much do I drink in everyday life"), symptom-monitoring diary |
| Module 3: Heart failure – how can physical activity help? (Physiotherapist, exercise therapists) | 60 | Physical activity recommendations for HF patients; experience with physical activity; individual ideas for physical activity after discharge; action and coping planning; action control | Interactive short lectures, partner work/group discussion, homework. Behavior change techniques [21]: 1, 21, 18, 5, 7, 8, 10, 16[a] | ppt-presentation, flipchart, educational booklet with HF information and worksheets ("my activity plan", "my activity record") |

| | | | | |
|---|---|---|---|---|
| Module 4: Heart failure – what changes in everyday life may arise? (Psychologist) | 60 | Illness burden and coping in everyday life; recognizing emotional distress, depression and anxiety; coping and seeking (professional) help; coping with sexual difficulties | Interactive short lectures, group discussion, individual work, relaxation exercise (optionally) | ppt-presentation, flipchart, educational booklet with HF information and worksheet ("peer stories") |
| Module 5: Heart failure – how can medicine help? (Physician) | 60 | HF medicines (main types, common side effects); medication adherence - unintentional and intentional barriers for taking medicine and corresponding management strategies; daily weight, pulse and blood pressure monitoring at home; physical activity plans; individual "take-home-messages" | Interactive short lectures, group discussion, individual work | ppt-presentation, flipchart, educational booklet with HF information and worksheets ("tips for taking medicine as prescribed", "my activity plan"), symptom-monitoring diary and worksheet ("my medication record") |

The detailed trainer manual is available in German and can be requested from the authors.

[a] 1 indicates "provide information on consequences of behavior in general"; 5, "goal setting (behavior)"; 7, "action planning"; 8, "barrier identification/problem solving"; 10, "prompt review of behavioral goals"; 16, "prompt self-monitoring of behavior"; 18, "prompt focus on past success"; 21, "provide instruction on how to perform the behavior".
